# Occurrence of anembryonic pregnancy with use of levonorgestrel subdermal implant (JADELLE®): a case report

**DOI:** 10.1186/s13256-018-1675-2

**Published:** 2018-04-29

**Authors:** Jude Moutchia-Suh, Dave-King Moutourou Mouemba, Calypse Asangbe Ngwasiri

**Affiliations:** 1 Bamenda Station Polyclinic, P. O. Box 457, Bamenda, North-West Region Cameroon; 2 Bamenda Regional Hospital, Bamenda, Cameroon; 3 Bamenjou District Hospital, Bamenjou, Cameroon; 4 Clinical Research Education, Networking & Consultancy (CRENC), Douala, Cameroon

**Keywords:** Progestin-only subdermal implant, JADELLE®, Anembryonic pregnancy, Case report, Africa

## Abstract

**Background:**

Progestin-only subdermal implants are one of the most effective contraceptive methods. Anembryonic pregnancy is not reported as a possible outcome in cases of contraceptive failure of these products. We present a rare case of anembryonic pregnancy occurring in a woman with levonorgestrel-releasing implant (JADELLE®).

**Case presentation:**

A 31-year-old Cameroonian (black African) housewife with a JADELLE® implant for 13 months, consulted at our hospital for a 1-month history of pelvic pain, prolonged menstrual bleeding, and spotting. She had a last normal menstrual period 8 weeks 1 day prior to presentation. On examination, there was suprapubic tenderness and blood trickling from her cervix. Despite a negative qualitative urine pregnancy test, an empty intrauterine gestational sac with mean sac diameter of 28 mm was visualized on pelvic ultrasound. Dilation and curettage with suction was done and she had complete relief from symptoms.

**Conclusion:**

This case report highlights the possibility of anembryonic pregnancy occurring in women using the levonorgestrel-releasing subdermal implant (JADELLE®).

## Background

A contraceptive implant is a common form of long-acting reversible contraception. Progestin-only subdermal implants (POSDIs) are very effective with failure rates of only 0.05% within the first year of typical use, providing prevention against pregnancy for 3 to 5 years [1, 2]. Contraceptive failures associated with POSDIs have been attributed to poor insertion technique, poorly timed insertion, drug interactions, product/method failure, and unknown factors [3, 4]. The levonorgestrel-releasing subdermal implant (JADELLE®) was approved by the Food and Drug Administration (FDA) in 1996 and is widely used in sub-Saharan Africa [2]. On the FDA’s and Bayer’s pharmaceutical product labels for JADELLE®, it is recognized that in case of contraceptive failure, ectopic pregnancy is a possibility [5, 6]. However, the possibility of occurrence of an anembryonic pregnancy in case of contraceptive failure with JADELLE® is not reported [5, 6].

Anembryonic pregnancy refers to a condition in which an embryo does not develop in a gestational sac [7]. A fertilized egg implants in the uterus, but the early embryo is resorbed, resulting in an empty gestational sac. Anembryonic pregnancy presents clinically with vaginal spotting or bleeding, with or without pelvic pain. Ultrasound findings typically reveal a mean gestational sac diameter (MSD) < 25 mm that shows no growth in 7–10 days, or MSD ≥ 25 mm without a detectable yolk sac [8]. We present a rare case of an anembryonic pregnancy occurring in a non-obese woman with JADELLE® implant. Diagnosis was initially missed, with presenting symptoms attributed to adverse effects of JADELLE®.

## Case presentation

A 31-year-old Cameroonian (black African) housewife with a JADELLE® subdermal implant in her left arm for 13 months, inserted by an experienced midwife, and a last normal menstrual period on T_0_ presented to our hospital on T_0_ + 8 weeks 1 day with complaints of pelvic pain, prolonged menstrual bleeding, and spotting of 1 month’s duration. The pain was insidious, cramping, had no aggravating/relieving factors, and was graded as 6/10 on a numerical rating scale. She used one mildly soaked sanitary pad daily and did not notice blood clots. Fourteen days (T_0_ + 6 weeks 1 day) after onset of symptoms, she consulted at a local health center where a beta-human chorionic gonadotrophin (hCG) qualitative urine pregnancy test was done with negative result. Her symptoms were attributed to adverse reactions to JADELLE® and she was prescribed paracetamol tablets. However, persistence of her symptoms prompted a second consultation at our hospital on T_0_ + 8 weeks 1 day.

In the past, she had five gestations: four normal vaginal deliveries at term at T_0_ - 10 years, T_0_ - 6 years, T_0_ - 4 years, and T_0_ - 2 years, and a premature stillbirth at T_0_ - 8 years. She was human immunodeficiency virus (HIV)-negative, had a single partner who was 35-years old, had no history of sexually transmittable infections, was on no routine medications, and denied alcohol consumption or tobacco smoking.

**Fig. 1:**
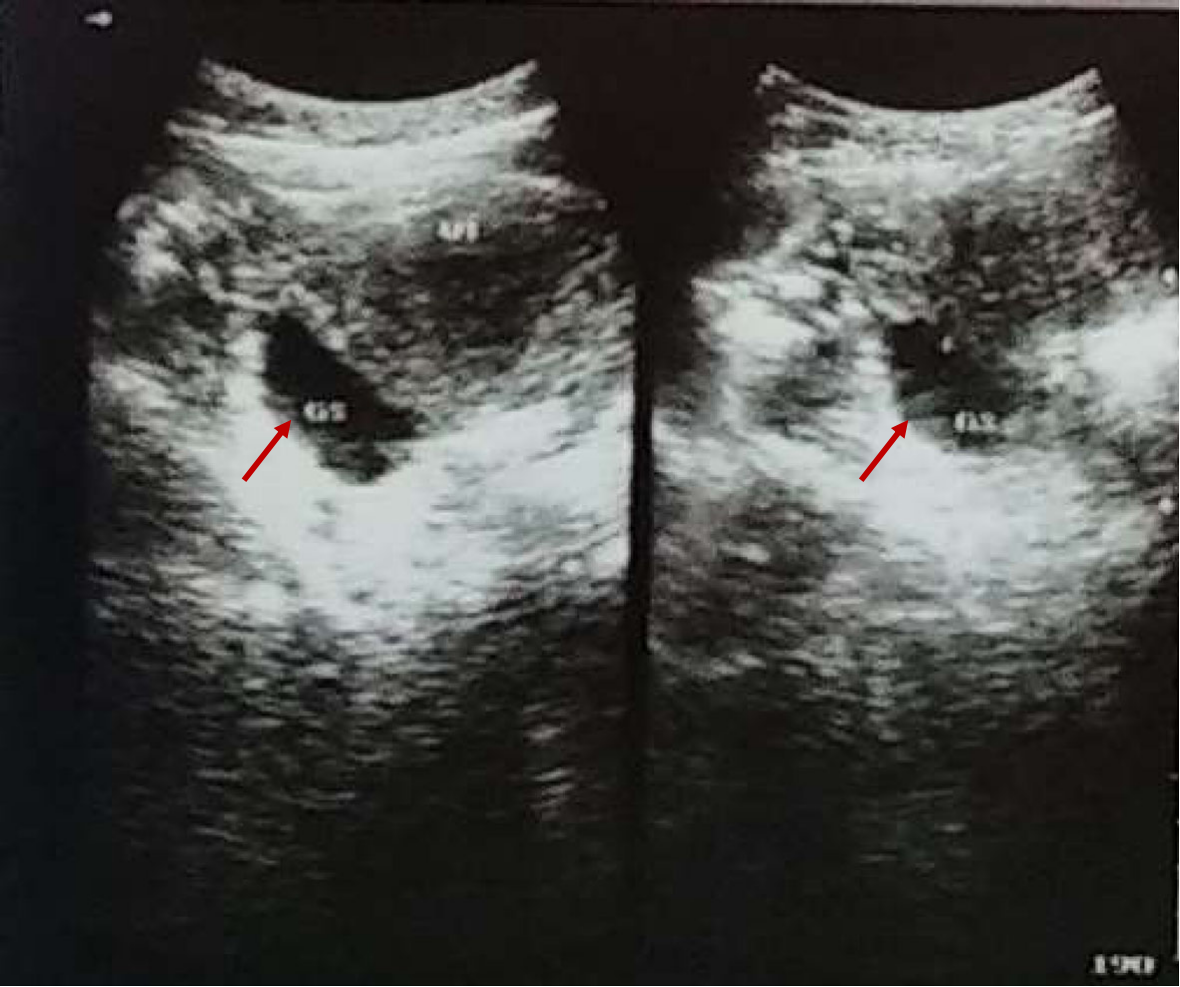
Pelvic ultrasound with arrows pointing to empty intrauterine gestational sac with mean sac diameter of 28 mm

**Fig. 2:**
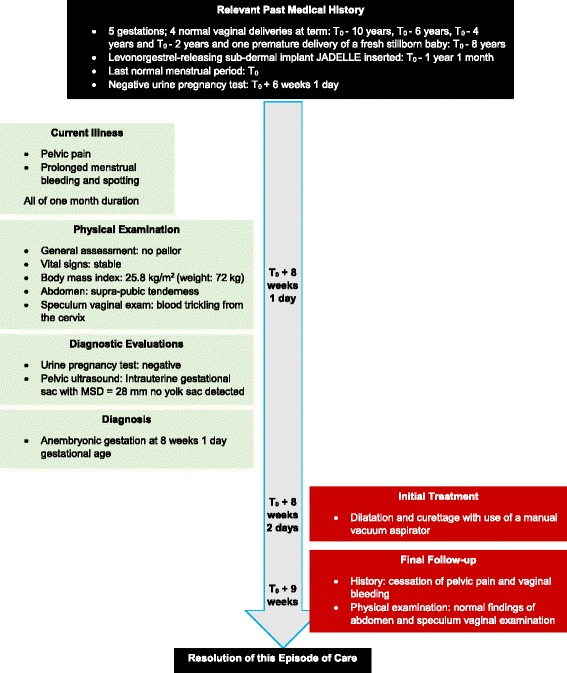
Case timeline. *MSD* mean gestational sac diameter

On physical examination, she was not pale, had stable vital signs and a body mass index of 25.8 kg/m^2^ (weight 72 kg). There was suprapubic tenderness and on speculum vaginal examination, there was blood trickling from her cervix. A repeat beta-human chorionic gonadotrophin (β-hCG) qualitative urine pregnancy test was negative. On pelvic ultrasound, by a radiologist, an intrauterine gestational sac with MSD of 28 mm was visualized, but a yolk sac was not detected (Fig. 1). We concluded on a diagnosis of an anembryonic pregnancy of 8 weeks 1 day gestational age (based on last menstrual period) and our patient was counselled and booked for dilatation and curettage. We performed a dilatation and curettage with suction the following day under local anesthesia, with aid of a manual vacuum aspirator. She was monitored for 6 hours and discharged home. Expelled products were not sent for histopathological analysis because of financial restrictions. During her follow-up visit 5 days later, she reported a cessation of pelvic pain and vaginal bleeding. A physical examination of her abdomen and speculum vaginal examination were normal (Fig. 2).

## Discussion

Levonorgestrel is a wholly synthetic and biologically active progestin that exhibits high progestational activity but no significant estrogenic activity. The levonorgestrel-releasing subdermal implant JADELLE® is a set of two flexible cylindrical implants, each approximately 43 mm in length and 2.5 mm in diameter with each containing 75 mg of the progestin. The total administered/implanted dose is therefore 150 mg. In the first month of use, the calculated mean daily release rate of levonorgestrel by the implants is approximately 100 μg/day [5, 6]. This declines to approximately 40 μg/day at 12 months, and to approximately 30 μg/day at 24 months and beyond [5, 6]. JADELLE® is gaining increasing popularity and usage in sub-Saharan Africa [2]. After insertion, mostly in the non-dominant arm, the implant protects against pregnancy for up to 3 to 5 years [2]. JADELLE® mediates contraception through: partial or complete inhibition of the gonadotropin surge thereby suppressing ovulation; increased cervical mucus viscosity thereby restricting the access of spermatozoa to the site of fertilization; and suppression of endometrial maturation thereby impairing implantation of the blastocyst [5, 6, 9]. It is a very effective means of contraception with a Pearl Index (pregnancies per 100 women-years) as low as 0.17 (95% confidence interval 0.04–0.30) during 5 years [6]. Although the absolute risk of ectopic pregnancy occurring with use of JADELLE® is reduced due to prevention of fertilization, JADELLE® is associated with increased relative risk of ectopic pregnancy in cases of contraceptive failure [5, 6]. In this case report, we describe a rare incident of anembryonic pregnancy following failed contraception with 13 months’ regular JADELLE® implant use. Due to the fact that an anembryonic pregnancy is not reported as a possible outcome in cases of contraceptive failure of JADELLE®, and because its clinical presentation overlaps with the common adverse reactions of JADELLE®, such as pelvic pain and prolonged menstrual bleeding and spotting, it was challenging to make an accurate clinical diagnosis. This diagnostic enigma was further compounded by two negative β-hCG qualitative urine pregnancy tests, done 2 weeks apart. A pelvic ultrasound scan was instrumental for an accurate diagnosis in this case.

HCG is secreted by the syncytiotrophoblast cells of the fertilized ova. β-hCG is detected in maternal serum or urine only after implantation and vascular communication between the syncytiotrophoblast and decidua, 8–10 days after conception. Qualitative pregnancy urine test strips commonly detect 25 mIU/ml hCG in urine [10]. In anembryonic pregnancy, levels of hCG are significantly lower compared to women with singleton pregnancies which progress until term [11]. It is therefore common to have a false-negative qualitative urine pregnancy test in anembryonic pregnancy.

We could not clearly establish the cause of contraceptive failure in this case. Reported causes of contraceptive failure associated with POSDIs include poor insertion technique, poorly timed insertion, and drug interactions [3, 4]. These were all ruled out in our case after a thorough history, and examination of the implant site. It is possible that this case of contraceptive failure resulted from product/method failure. JADELLE® inhibits ovulation in approximately 45–85% of menstrual cycles [12]. In a 5-year multicenter randomized clinical trial involving 600 women on JADELLE®, with 272 women completing the 5 years, three women became pregnant [13]. Out of these three women, one of them had an ectopic pregnancy [13]. It is likely that synthetic progestins increase the risk of a fertilized ovum implanting out of the uterus by impairing fallopian tube ciliary function [14]. However, the effect of these synthetic progestins on anembryonic pregnancy has not been clearly elucidated. Out of 27,758 females with an etonogestrel-releasing subdermal implant reporting adverse reactions to the FDA, 9 (0.03%) reported having anembryonic pregnancy, with a majority being 30 to 39-years old with 2 to 5 years of regular implant use [15]. Anembryonic pregnancy is typically caused by chromosomal abnormalities from a poor quality ovum or sperm, abnormal cell division in the embryo following fertilization, and/or possible exposure to teratogens. Our patient’s partner was not of an advanced age, and we did not identify any exposure to a possible teratogen in our patient’s history. Synthetic progestins decrease production of natural progesterone from the ovary. Natural progesterone plays a role in oocyte maturity and embryo development [16]. Although immature oocytes can be normally fertilized, the developmental capability of the resultant embryo is reduced, compared to embryos resulting from mature oocytes [17]. Also, synthetic progestins create a poor environment for the development of the implanted embryo by suppressing endometrial maturation. More research is required to explicate the influence of synthetic progestins on anembryonic pregnancy.

## Conclusions

This case report highlights the possibility of an anembryonic pregnancy occurring in women with levonorgestrel-releasing subdermal implant JADELLE®. While further research is necessary to explicate the influence of POSDIs on anembryonic pregnancy, proper evaluation of these women in the event of pelvic pain, prolonged menstrual bleeding, and spotting is imperative. Ultrasound scans are instrumental in these evaluations.

## Patient’s perspective

Our patient’s opinion focused on the fact that she chose JADELLE® because it is efficient, reversible, convenient, and has bearable side effects. She understood her symptoms as adverse reactions to JADELLE®, and despite being aware of the possibility of having an ectopic pregnancy in failed contraception, she was not aware of the possibility of having an anembryonic pregnancy. She was satisfied with the management of the case and expressed the desire to inform other women on JADELLE® of the possibility of having an anembryonic pregnancy.

“After my fifth pregnancy, my husband and I decided to halt child bearing. Among the wide range of contraceptive options proposed to us, we chose JADELLE® because it was most efficient, reversible, and convenient with tolerable side effects. Use of JADELLE® for 13 months was uneventful until last month when I had pelvic pain and prolonged menstrual bleeding, with drops of blood on my sanitary pad. I immediately knew these symptoms were side effects of JADELLE® and I visited the center where the product was inserted. After discussing with the midwife, she confirmed that these symptoms are normal with use of JADELLE®. She decided to rule out a pregnancy with a urine pregnancy test, and this came back negative. I went back home satisfied, but due to persistence of pelvic pain and having vaginal bleeding for 2 weeks, I decided to consult at a different hospital. Here, a urine pregnancy test was still negative and the doctor recommended an ultrasound. I was perplexed when he told me I was pregnant and had an empty sac in my womb. This was scary at first, but I was reassured after the doctor explained the condition and management options to me. I did not want to keep the sac any longer, so I instructed him to do a dilatation and curettage. I felt light pain during the procedure, but a few days after the procedure, I no longer felt pain and vaginal bleeding stopped. Overall, I am satisfied with the management of my case and have regained normal health. I had been counselled on the possibility of having an ectopic pregnancy if JADELLE® fails, but I was not aware I could also have an empty sac in my womb. I am now aware of this possibility and will be happy if other women on JADELLE® implant also become aware of this possibility.”

## Abbreviations

FDA: Food and Drug Administration
hCG: Human chorionic gonadotrophin
MSD: Mean gestational sac diameter
POSDIs: Progestin-only subdermal implants
